# Building Cross-sectoral Collaborations to Address Perinatal Health Inequities: Insights From the Dutch Healthy Pregnancy 4 All-3 Program

**DOI:** 10.34172/ijhpm.8115

**Published:** 2024-07-09

**Authors:** Leonie A. Daalderop, Lisa S. Barsties, Frank van Steenbergen, Adja J.M. Waelput, Jacqueline Lagendijk, Jasper V. Been, Eric A.P. Steegers, Derk Loorbach

**Affiliations:** ^1^Department of Obstetrics and Gynaecology, Erasmus MC, University Medical Centre Rotterdam, Rotterdam, The Netherlands; ^2^Dutch Research Institute for Transitions, Erasmus University Rotterdam, Rotterdam, The Netherlands; ^3^Division of Neonatology, Department of Paediatrics, Erasmus MC – Sophia Children’s Hospital, University Medical Centre Rotterdam, Rotterdam, The Netherlands; ^4^Department of Public Health, Erasmus MC, University Medical Centre Rotterdam, Rotterdam, The Netherlands

**Keywords:** Cross-sectoral Collaborations, Perinatal Health Inequities, Facilitators and Barriers, Transition, Action Research

## Abstract

**Background:**

Addressing perinatal health inequities is the joint responsibility of professionals working for local governments, the medical, social, and public health sector. Cross-sectoral collaboration between these professionals is challenging. For such collaborations to succeed, a transition, ie, a fundamental shift in the dominant structure, culture, and practices at the systems level, is necessary. We investigated facilitators and barriers for cross-sectoral collaborations, when addressing perinatal health inequities in the Netherlands. Additionally, we studied how cross-sectoral collaborations can be facilitated by action research.

**Methods:**

We used interview and questionnaire data of the Healthy Pregnancy 4 All-3 (HP4All-3) program, which resulted from action research in six Dutch municipalities. All interviews were coded using open codes related to facilitators and barriers for cross-sectoral collaboration and categorized into three subgroups: structural, cultural, or practical. The answers to the questionnaire were analyzed and summarized quantitatively.

**Results:**

We conducted 53 interviews with a total of 81 professionals. The most important ingredients for cross-sectoral collaborations mentioned by the interviewees were: (1) structural: having a solid network with a clear overview of professionals working in the different sectors, (2) cultural: having a joint vision/goal, and (3) practical: short lines of communication and timely sharing of information. A total of 85 professionals filled in (parts of) the questionnaire. Two-thirds to over 80 percent replied that the HP4All-3 program had an added value in building cross-sectoral collaborations.

**Conclusion:**

Our research shows that cross-sectoral collaborations in the context of perinatal health are hampered by structural, cultural, and practical barriers. Analyzing facilitators and barriers at these three levels helps to identify bottlenecks in cross-sectoral collaboration. Action researchers can be of great advantage in facilitating collaboration, as they can offer an open setting for reflection and instigate a sense of urgency for building collaborations.

## Background

**Key Messages**

**Implications for policy makers**

- The determinants of perinatal health inequities are interlinked and both medical and social. Addressing these determinants requires new forms of collaboration involving a range of professionals working for local governments, and in the medical, social, and public health sector. This requires institutional changes to facilitate professionals to work in collaboration on addressing the root causes of perinatal health inequities.
- Building stable cross-sectoral collaborations to address perinatal health inequities requires (1) a solid network with a clear overview of professionals working in different sectors, (2) a joint vision/goal within the defined network, and (3) short lines of communication.
- Action researchers can play a crucial role in overcoming structural barriers to cross-sectoral collaboration as they can initiate a change process by offering an open setting for reflection and instigating a sense of urgency to tackle persistent societal problems.

**Implications for the public**

Cross-sectoral collaboration is a promising way to address persistent societal problems, such as inequities in perinatal health. The determinants of these inequities are interlinked and both medical and social, necessitating collaboration between professionals working for the local government, the medical, social, and public healthcare sector. Building such cross-sectoral collaborations requires intrinsically motivated leaders who can create a sense of urgency, bridge sectors, and facilitate communication. Additionally, establishing a common understanding and agreement on the issue at stake is crucial. All sectors involved should align their objectives to create a unified vision that reflects the shared interests and desired outcomes. This can be facilitated by building trust among the involved professionals, by focusing on transparent and open communication, as well as a clear division of roles and responsibilities to avoid confusion and ensure accountability. External parties, such as action researchers, can help to initiate meetings, where professionals can get to know, enthuse, and motivate each other.

Large health inequities exist between and within cities across high-, middle-, and low-income countries.^1-7^ Substantial inequities are already present during pregnancy and the perinatal period, which have major implications for public health as they constitute developmental origins of socioeconomic variation in adult health and disease.^8-13^ The Dutch perinatal mortality and morbidity rates were among the highest in Europe and had a slow temporal decline compared to other European countries.^14^ These adverse perinatal health outcomes were particularly observed in deprived areas of the Netherlands.^3,15-17^ Research showed that next to the negative impact of well-known medical and obstetric risk factors, risk accumulation of non-medical factors related to a person’s socioeconomic status (SES) and physical environment underlie many of the health inequalities at birth.^4,18-21^ Next to the direct negative health effects of having a low SES, living in deprivation can be an overwhelming source of chronic stress which is a serious health risk factor. Addressing the non-medical risk factors associated with these inequities, falls under the responsibility of a range of professionals working for local governments, as well as those working in the medical, social, and public health sector. This is promoted by the “Health in All Policies” framework which acknowledges the interconnectedness of health with social, cultural, environmental, and economic factors and therefore calls for cross-sectoral responses.^22^ Cross-sectoral collaboration is defined as the “linking or sharing of information, resources, activities, and capabilities by organizations in two or more sectors to achieve an outcome that could not be achieved by organizations in one sector separately.”^23^ Such a cross-sectoral approach provides a foundation for policy-makers and professionals in the medical, social, and public health sector to collaborate and to establish integrated medical and social care. Although it is considered a promising approach to societal challenges and existing inner- and intersectoral fragmentation, the development and implementation of cross-sectoral collaborations often prove difficult in practice.^23^ Therefore, a transition is required.

 A transition is a non-linear, structural shift in a subsystem’s dominant structure, culture, and practices (ie, its regime).^24^ A subsystem’s structure relates to its organization, budgets, and regulations. Culture can be defined by a subsystem’s set of shared values and perceptions. Practices refer to professionals’ behavior, actions, and routines in daily practice. Structures, cultures, and practices are path dependent because of interests, made investments, and established positions. The default option for professionals is to continue in the same direction. This becomes problematic when the context changes and society starts to place new demands upon the regime. In healthcare, for example, the regime has historically developed based on specialization, efficiency, and cure of diseases. However, when research on the relatively high perinatal mortality and morbidity rates in the Netherlands was published, the Ministry of Health, Welfare and Sport started to ask for more prevention, integrated solutions, and social interventions to improve public health.^25^ This places demands that the current healthcare regime cannot meet. To better understand, guide, and accelerate a desired transition, action research is a useful tool. Action research is an umbrella-term for various research processes and methods that, notwithstanding their diversity, always consist of research, participation, and action.^26^ Action research can facilitate the coproduction of new ideas, practices, and collaborations with participants from different sectors and thereby support a desired transition at the local level.^27^

 In the Netherlands, several local and national intervention programs were developed with the overarching aim to address health inequities from birth onwards (Table 1).^28-30^ These programs tested and implemented several interventions throughout the perinatal healthcare system. The perinatal healthcare system encompasses care for women and their families during the preconception, prenatal, postpartum, and early childhood periods (ie, the first 1000 days of life). As these periods bear substantial plasticity, they enable improvement via early interventions that help to develop the functional capacity of a child to respond to health challenges throughout life. Over time, moving from Ready for a Baby to the second Healthy Pregnancy 4 All-3 (HP4All-3) program, learning took place with regards to perinatal health inequities’ underlying root causes and the need to address the wider societal context within which they are (re)produced. Building on these insights, the HP4All-3 program (2018-2021) was developed. The overarching aim of HP4All-3 was to study and explore drivers for transformative change in institutional structures, culture, and practices towards the implementation of perinatal health into municipal approaches and policies concerning health inequities resulting in a cross-sectoral approach to perinatal health. This has been done by conducting action research in six Dutch municipalities.^31^ Additionally, the results of the HP4All-3 action research were used to inform a knowledge dissemination program on the need to implement perinatal health into municipal approaches and policies concerning health inequities. This knowledge dissemination program was rolled out among the 156 municipalities with the highest share of disparities out of all 380 Dutch municipalities (2018).

**Table 1:** Overview of Research Programs Aimed at Addressing Perinatal Health Inequities Anteceding the Healthy Pregnancy 4 All-3 Study

**Program**	**Initiator(s)**	**Financier**	**Time Period**	**Location**	**Key Approaches**
Ready for a Baby^28,32^	Erasmus MC, GGD Rotterdam-Rijnmond^a^	Municipality of Rotterdam	2008-2012	City of Rotterdam^b^	Health promotion through customized preconception care Systematic antenatal risk assessment (R4U^c^) with increased attention for non-medical risk factors Interdisciplinary risk-directed care Establishment of a primary birth care center in the Erasmus MC (Rotterdam)
Healthy Pregnancy 4 All-1^29,33,34^	Erasmus MC	Ministry of Health, Welfare, and Sport	2011-2014	14 Dutch municipalities^b^: Almere, Amsterdam, Appingedam, Delfzijl, Enschede, Groningen, Heerlen, Menterwolde, Nijmegen, Pekela, Schiedam, The Hague, Tilburg, Utrecht	Health promotion through customized preconception care Antenatal R4U risk assessment followed by patient-tailored multidisciplinary care pathways
Healthy Pregnancy 4 All-2^30,35-37^	Erasmus MC	Ministry of Health, Welfare, and Sport	2014-2017	10 Dutch municipalities^b^: Almere, Amsterdam, Arnhem, Dordrecht, Groningen, Rotterdam, Schiedam, The Hague, Tilburg, Utrecht	Structured risk assessment during pregnancy and customized maternity care Interconception care through PCHC Optimizing postnatal R4U risk assessment in PCHC

Abbreviation: PCHC, preventive child healthcare. ^a^GGD Rotterdam-Rijnmond provides the Municipal Health Services for the municipality of Rotterdam as well as for the surrounding municipalities. ^b^All municipalities were selected based on their relatively poor perinatal and child health outcomes. ^c^R4U stands for Rotterdam Reproductive Risk Reduction and is a 70-item score card, assessing risks for adverse pregnancy and child health outcomes in six domains (social status, ethnicity, care, lifestyle, medical history, and obstetric history).^38^

 Here, we report our investigation of structural, cultural, and practical facilitators and barriers for cross-sectoral collaboration to address perinatal health inequities, using data of the HP4All-3 program. Additionally, we studied how cross-sectoral collaboration can be facilitated by action research.

## Methods

###  Design 

 Within the HP4All-3 action research, we carried out a mixed method approach, which included a desk study, interviews, interactive group sessions, and a questionnaire. We used an explanatory sequential design in which we first collected quantitative data through a desk study, which was used to inform the qualitative part of the study (ie, interviews and group sessions). For a detailed description of the HP4All-3 study design, we refer to the study’s protocol.^31^ For the present study, we used data from the desk study, the interviews, and the questionnaire (See Data Collection).

###  Participating Municipalities and Stakeholders 

 We hypothesized that our research would have the biggest impact in municipalities where inequities are most severe. We consider awareness a key ingredient for building cross-sectoral collaboration networks, as it serves as a catalyst for action by bringing attention to the issue at stake, mobilizing support, promoting behavioral change among professionals, and empowering individuals with knowledge and skills to foster change in daily practice. We combined the evidence of the existence of persistent inequities in specific municipalities with the assumption that awareness to address these inequities would be high, therefore allowing for a more effective participation and impact thereof. Based on this, we decided to conduct our research in municipalities where perinatal health inequities are most severe.

**Figure 1:**
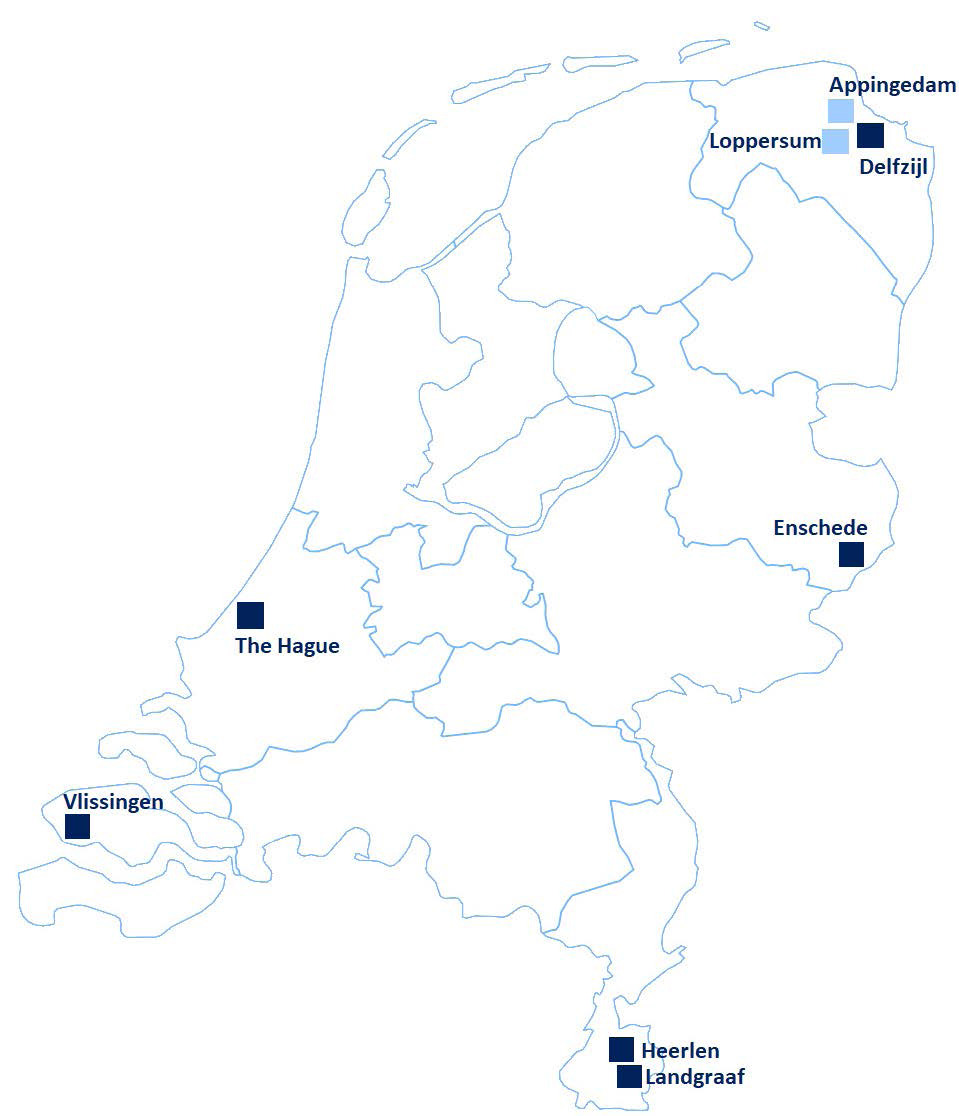
Geographical Location of the Participating Municipalities. * The HP4All-3 research took place in the year before the municipality of Delfzijl was supposed to merge with two neighboring municipalities, Appingedam and Loppersum, into the municipality Eemsdelta. Therefore, we decided to include Appingedam and Loppersum in addition to Delfzijl. As such, these three municipalities were examined as one case.

 Following a baseline measurement, municipalities with a relatively high incidence of adverse perinatal health outcomes (ie, preterm birth and small for gestational age [SGA]), a high proportion of children living in families on welfare, and a low municipal SES were selected for participation. Data on adverse perinatal health outcomes was obtained at the municipal level (2011-2015) from the Netherlands Perinatal Registry (Perined, https://www.perined.nl/). Data on the proportion of children living in families on welfare over the year 2015 was used as a proxy for children living in poverty. This data is freely accessible on the website https://www.waarstaatjegemeente.nl/. Municipal SES scores were derived from neighborhood SES scores over the year 2016, which are openly accessible on the website of the Netherlands Institute for Social Research (SCP, https://www.scp.nl/). SCP neighborhood SES scores are based on (1) mean annual income per household, (2) percentage of households with a low income, (3) percentage of households with a low level of education, and (4) percentage of unemployed inhabitants. These scores were weighted by the number of inhabitants in the neighborhood and summed for each municipality. This resulted in an overarching municipal SES score. A higher SES score indicated a more affluent municipality. Municipalities with a SES score within the lowest quintile were considered as having a low SES. In addition to these criteria, we assessed the extent to which municipalities were already addressing perinatal health inequities. To do so, we developed a search strategy for Google, using free text terms related to perinatal health, pregnancy, and municipal documents (eg, policy documents and budget overviews) to find information concerning municipal policies, activities, and cross-sectoral collaborations directed at addressing perinatal health inequities at the local level. The combination of these criteria resulted in the selection of six municipalities with significant inequities, of which three had less than 70 000 inhabitants and three had more; four of these municipalities were already active in addressing perinatal health inequities and two municipalities were not (based on the Google search) (Table 2). Figure 1 shows the location of the participating municipalities within the Netherlands. Detailed information regarding the selection procedure of municipalities can be found in the HP4All-3 study protocol.^31^

**Table 2:** Characteristics of Participating Municipalities

**Municipality**	**Inhabitants**^a^	**Location**^b^	**Perinatal Health Approach**^c^	**Perinatal Health Indicators**		**Children on Welfare (%)**^f^	**Interviewees**		**Questionnaire**	
				**Perinatal Mortality**^d^	**PTB and/or SGA**^e^		**Total**	**Sectors**	**Total**	**Sectors**
Eemsdelta	45 587	Northern	No	4.8	143.8	10.0	12	Municipal: 4 Medical: 5 Social: 0 Public: 3	9	Municipal: 2 Medical: 4 Social: 0 Public: 3
Enschede	159 732	Northern	No	4.3	175.2	10.0	11	Municipal: 3 Medical: 4 Social: 2 Public: 2	10	Municipal: 2 Medical: 2 Social: 4 Public: 2
Heerlen	86 936	Southern	Yes	4.0	206.5	13.0	18	Municipal: 5 Medical: 10 Social: 0 Public: 3	16	Municipal: 5 Medical: 7 Social: 1 Public: 1
Landgraaf	37 262	Southern	No	3.9	206.8	8.0	19	Municipal: 6 Medical: 9 Social: 1 Public: 3	5	Municipal: 3 Medical: 2 Social: 0 Public: 0
The Hague	548 320	Central	Yes	5.1	179.1	11.0	12	Municipal: 5 Medical: 5 Social: 0 Public: 2	12	Municipal: 2 Medical: 9 Social: 0 Public: 1
Vlissingen	44 358	Southern	Yes	5.4	176.6	10.0	9	Municipal: 4 Medical: 2 Social: 1 Public: 2	7	Municipal: 3 Medical: 1 Social: 0 Public: 3
Netherlands	17 475 415	-	-	4.5	157.5	6.0	-	-	-	-

Abbreviations: PTB, preterm birth; SGA, small for gestational age. ^a^Number of inhabitants on January 1, 2021; ^b^Location refers to the location of the participating municipality in the Netherlands and is subdivided into northern, central, and southern Netherlands; ^c^Shows if participating municipalities were already active in addressing perinatal health inequities based on the findings of the Google search and additional information gained through the interviews; ^d^Perinatal mortality was defined as death occurring between 24 weeks of gestational age and 7 days after birth expressed per 1000 births; ^e^ SGA defined as a birth weight below the 10th centile adjusted for gestational age expressed per 1000 births and PTB was defined as delivery of a liveborn baby before 37 completed weeks expressed per 1000 births. All perinatal health indicators refer to 2015-2019; ^f^Percentage of children up to the age of 18 years who live in a family on welfare in 2020.

 We pre-specified a list of key stakeholders that generally should be involved in building cross-sectoral collaborations to address perinatal health inequities. This list was based on expert meetings within our research group. The defined key stakeholders include professionals working for the local government (ie, municipality) and professionals working in the medical, social, or public health sector.

 By local government we mean municipalities. Municipalities are the third tier of the Dutch government, after the national government and the provinces. There are 352 Dutch municipalities (2021), where mayors, aldermen, and civil servants are involved in policy-making. We assume that civil servants and aldermen from the fields of welfare, work and income, youth, and public health can play an important role in facilitating the support of families living in precarious conditions by developing policy programs that are, either directly or indirectly, aimed at addressing perinatal health inequities. The municipal government is in contact with its inhabitants through municipal services, such as public libraries, schools, police, etc.

 The medical sector consists of all professionals and institutions that are part of the healthcare system. Within the HP4All-3 program, we focused on professionals who are involved in the care for parents(to-be) and/or their children during the first 1000 days of life (ie, the preconception, prenatal, postpartum, and early childhood period up to the age of 2 years). These professionals include general practitioners (GPs), midwives, obstetricians, maternity care assistants, and pediatricians.

 The social sector includes all professionals and organizations that are operational in the fields of social support, youth care, and societal participation. Examples of such organizations are: welfare and mental health organizations, as well as neighborhood teams. In 2015, the Dutch national government decentralized social and healthcare responsibilities to municipal governments. The decentralization has led to the development of neighborhood teams as to provide integrated tailor-made support to inhabitants of neighborhoods, aimed at, for instance, detecting parenting problems at an early-stage. In 2019, 83% of Dutch municipalities had at least one neighborhood team. Although the composition of neighborhood teams can differ per municipality, they often consist of social support consultants, social workers, mental healthcare professionals, and youth workers.

 The public health sector is a rather broad sector. Within the HP4All-3 study, we focused on organizations that are involved in the health and development of children, such as Preventive Child Healthcare (PCHC) organizations, which monitor children from the first week of life until the age of 19 years. PCHC consultations and services are free of charge and include vaccinations, growth and development measurements, advice on health and health behavior, and, if needed, referral to specialized care.^39^ Between 2011 and 2013, PCHC consultations at different age points had an attendance rate of approximately 95% for children between zero and four years old across the country.^40^

###  Data Collection 

####  Interviews

 For the interviews, we selected professionals based on the predefined list of key stakeholders. For each participating municipality, we aimed to interview two professionals from each of the following stakeholder groups: local government, medical, social, and public health sector. For the local government (ie, municipality), we sought to interview aldermen or civil servants working in the policy domain of welfare, work and income, youth, or public health. For the medical sector, we aimed to include at least one professional working in a local hospital (ie, obstetrician, midwife, or pediatrician) and one professional working outside the hospital (ie, GP, primary care midwife, or maternity care assistant). Regarding the social sector, we planned to include two professionals from a neighborhood team, social welfare organization, or social workers, depending on the local situation. Lastly, regarding the public health sector, we intended to include two professionals working for a PCHC organization. To do so, we searched the websites of the participating municipalities, local hospitals, midwifery practices, maternity care organizations, GP practices, welfare organizations, etc., to identify potentially eligible interviewees. Additionally, we applied snowball sampling by asking participants whom we already interviewed about other potentially eligible interviewees in addition to our pre-specified list of key stakeholders. To guide the interviews, we developed an interview protocol (Supplementary file 1), which was tested in advance. Between January and September 2019, we conducted seven to 12 semi-structured interviews per municipality, with one to three respondents per interview. All interviews were conducted face-to-face or by telephone by two researchers of the HP4All-3 research team (alternately LAD, LSB, and FvS). All interviews were audio-recorded and lasted approximately one hour.

####  Questionnaire 

 As a follow-up on the interviews and group sessions (for a description of the group sessions and their results see Barsties et al^41^), a questionnaire (Supplementary file 2) was distributed in March 2021. The aim of this questionnaire was to determine whether our study, consisting of interviews and group sessions, has contributed to the (further) development of cross-sectoral collaborations in the participating municipalities. The questionnaire was distributed via email among all interviewees and participants of the group sessions, in which both interviewees as well as other interested and change-inclined professionals participated.

###  Analysis 

 All interviews were transcribed verbatim and were coded by two researchers (LAD and LSB), using open codes related to facilitators and barriers for cross-sectoral collaboration. To increase intercoder reliability, 15% of the interviews were coded by both researchers in an iterative process. After double coding 5% of the interviews, LAD and LSB discussed existing differences and proceeded with the next 5% of interviews. After the third round, no differences between the codes were found. All facilitators and barriers were categorized into three subgroups: structural, cultural, or practical. Structural facilitators/barriers relate to organizational structures, budgets, and regulations of professionals/organizations. Cultural facilitators/barriers are defined as (mis)understandings due to cultural similarities or differences based on views, values, and paradigms. Practical facilitators/barriers are factors that relate to the behavior, actions, and routines of professionals.

 The different facilitator and barrier codes were merged into larger themes. These themes were analyzed at the interviewee level as well as at the group level, using three different subgroups: (1) municipalities with <70 000 inhabitants vs. municipalities with >70 000 inhabitants, (2) municipalities that were already active in addressing perinatal health inequities vs. municipalities that were not yet active, (3) type of professional (professionals working for the local government, in the medical, social, or public health sector). The answers to the questionnaire were analyzed and summarized quantitatively.

## Results

 We conducted 53 interviews with a total of 81 people, varying from nine to 19 interviewees per participating municipality. Of these, 35 worked in the medical sector, 27 for local governments, 15 in the public health sector, and 4 in the social sector (Table 2). The interviews yielded a wide range of facilitators and barriers, of which the five most frequently mentioned are described in the sections below, categorized by subgroups. As facilitators and barriers are directly linked, meaning that facilitators often are the inverse of barriers and vice versa, we report them without making a strict distinction between these two.

###  Structural Facilitators and Barriers 

 We identified four structural facilitators and/or barriers: having a solid network, fragmentation, not knowing and/or finding each other, and finances.

**Figure 2:**
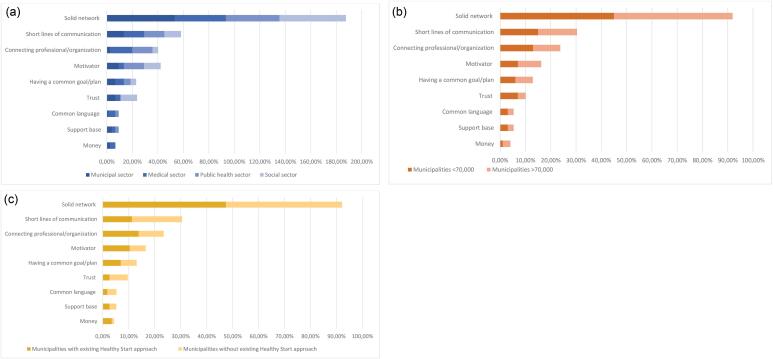
Sum of the Percentage of Respondents Within the Specified Groups Who Mentioned a Facilitator. Figure 2a shows mentioned facilitators by type of professional, 2b by municipal size, and 2c by municipal activity. The percentages of the different groups are added together, so that the total percentage can exceed one hundred.

 Solid network: Having a clear overview of professionals working in the different sectors facilitated collaboration. A clear overview is created through regular (network) meetings between professionals, which enable them to get to know each other and to gain insight into the work of others: *“Our awareness is often limited to our direct collaboration partners. What strikes me is the more meetings there are, the more initiatives emerge” *(alderman youth and education).Not having a predefined agenda is conducive to getting to know each other better, as this provides room for joint reflection and brainstorming. Across the three different subgroups, having a solid network was the most mentioned facilitator for cross-sectoral collaboration (Figure 2). This theme consisted of 11 codes, which are summarized in Supplementary file 3.

 Fragmentation: Fragmentation was described as a major cause of ineffectiveness and inefficiency, contributing to the lack of a clear overview of professionals. Fragmentation existed on several levels: in municipalities, sectors, and organizations. Fragmentation is perpetuated by professionals who prefer to work alone: *“Egos really are a problem. We all consider ourselves experts but don’t dare to connect with professionals from other sectors. Admitting that you failed and learning from it together takes guts” *(manager of a youth care organization).Additionally, some professionals mentioned that it is important that all involved partners are motivated to collaborate and convinced that collaborating has an added value.

**Figure 3:**
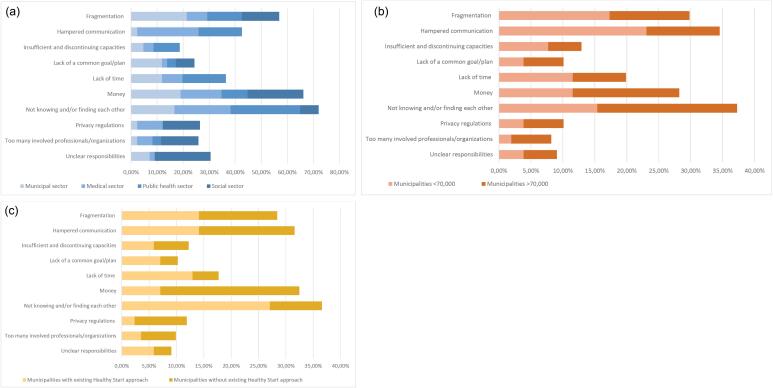
Sum of the Percentage of Respondents Within the Specified Groups Who Mentioned a barrier. Figure 3a shows mentioned barriers by type of professional, 3b by municipal size, and 3c by municipal activity. The percentages of the different groups are added together, so that the total percentage can exceed one hundred.

 Not knowing and/or finding each other’s work: This entails not only the inability to practically get in touch with other professionals due to physical distance or lacking contact details, but also the unfamiliarity with the work of professionals from other sectors and their asset to one’s own work. For example, one of the respondents mentioned: *“We as professionals cannot find each other and don’t even know what other professionals actually do. This means that we are less likely to think of them as potential collaboration partners. It’s easier to pick up the phone if you’ve seen or know someone” *(PCHC professional).Regardless the sector professionals work in, the size of the municipality, or a municipality’s (in)activity in addressing perinatal health inequities, not knowing and/or finding each other was the most frequently mentioned barrier (Figure 3).

 Finances: Not being able to finance the network, structure, and activities necessary for cross-sectoral collaboration is experienced as a barrier. A professional working for a youth care organization stated: “*The problem is not that people don’t want to collaborate, but that everyone has to do so much already and that there is only little money. That means that you have to make choices.”* Also, temporary financial structures lead to the discontinuation of otherwise successful collaborations. Clear financial structures are a prerequisite for cross-sectoral collaborations, as to secure that all involved partners receive the financial reward they agreed upon. Finally, flawed financial incentives, such as postponing the referral of patients so that the care provider in question can make a financial claim him/herself, make it difficult to work cross-sectorally.

###  Cultural Facilitators and Barriers 

 We identified two cultural facilitators and/or barriers: having a shared goal/plan and having a person who motivates others to collaborate.

 Having a shared goal/plan: When collaborating across sectors, it is important to formulate a shared vision and goal/plan, including a clear division of roles. If involved professionals do not know exactly what others are working on, it is hard to agree on plans and actions to be taken and stay motivated. This has been summarized by a civil servant in the field of societal development: *“You need to clearly agree on plans and actions to be taken: who is doing what, what are our goals, and how do we reach them? That makes it more concrete, because just having the will to do something is not enough.*”

 Motivator: The availability of a frontrunner, who has great affinity with the topic on which collaboration should take place, who continuously emphasizes the importance of collaboration, and who organizes meetings to encourage stakeholders to collaborate is experienced as an important facilitator. A project manager at a PCHC organization stated: “*You need a frontrunner. You need people that go all out. People that say: we sit and talk about it. People who say: this is important. People who say: this is part of your job*.”

###  Practical Facilitators and Barriers 

 Four practical facilitators and barriers were mentioned by the participants: having a connecting professional/organization, short lines of communication, hampered communication, and lack of time.

 Connecting professional/organization: Many professionals described that a contact person or case manager who knows the key stakeholders from different sectors facilitates collaboration. If there is no clear contact person or case manager, the presence of care pathways can also help to find the right professionals. One of the respondents mentioned: *“We have developed social care pathways throughout the city to clarify whom to approach in case of a patient being in trouble. We have many neighborhood teams in the city and these care pathways really help to find and approach them” *(medical advisor/gynecologist).

 Short lines of communication/hampered communication: Having short lines of communication with professionals working in different sectors facilitates collaboration. Working in close proximity to each other, eg, in the same building, can help to establish these short lines. For example, a PCHC professional mentioned: *“Recently, we started working in a midwifery practice one day a month. Although this is a rather new development, we already noticed that this makes communicating easier.” *On the other hand, information that is not being shared or shared too late between professionals makes it hard to collaborate. Communication is also challenging as professionals from different sectors lack a shared vocabulary; they simply do not understand each other. Lastly, different organizational cultures, such as working hours and perceptions of what is part of one’s job, hamper a smooth communication between sectors: “*It’s the language. I think that professionals working in the medical sector think very differently about meetings. They don’t consider them productive working hours. While professionals working for the local government or in the social sector are used to have a lot of meetings on a daily basis, that’s their job” *(professional working in higher vocational education for midwives).

 Lack of time: Many professionals find it difficult to collaborate across sectors as they feel there is no time for it next to what they consider to be the core tasks and responsibilities of their everyday work. Especially GPs were mentioned to be professionals that are being approached for many projects and collaborative structures, making it impossible for them to collaborate in all of them. When asked what makes cross-sectoral collaboration hard, a pediatrician answered: “*We would like to participate in every network meeting. But these meetings often last for two hours. That’s simply not possible. Therefore, we have to make choices*.”

 In addition to these 10 facilitators and barriers other facilitators and barriers were mentioned. They are described in Supplementary files 4 and 5.

###  Action Research and Cross-sectoral Collaborations

 A total of 85 professionals filled in (parts of) the questionnaire. Between municipalities, the responses varied from five to 16. The questionnaire was completed by 25 professionals working in the medical sector, 18 professionals working for the municipal government, 20 professionals working in the public health sector, and 5 professionals working in the social sector (Table 2). The response rate differed per question as questions were not mandatory to complete.

 Several questions related to the contribution of the HP4All-3 program to the process of building cross-sectoral collaborations. From the professionals who answered these questions (n = 35-37; 41%-44%), two-thirds to over 80% replied that the HP4All-3 program had an added value (Table 3). The HP4All-3 program especially helped in clarifying the urgency of enabling a healthy and promising start. A civil servant observed that “*during the group sessions, connection between the social and the medical sector was created. It had a connecting effect to understand the urgency of this topic in a co-creative setting.*” Participating in the HP4All-3 program also affected local developments concerning the topic of a healthy and promising start in general. Approximately 60% of the respondents indicated that participation in the HP4All-3 program helped in finding/reaching professionals from other sectors, starting new collaborations, and improving existing ones. This has been summarized by a PCHC professional who stated that “*collaboration with other professional groups improved [since participation in the HP4All-3 program]. We know each other better and know how to find each other. New initiatives always motivate and enthuse.*”

**Table 3:** Overview of Respondents’ Experienced Contribution of the Healthy Pregnancy 4 All-3 Program to the Process of Building Cross-sectoral Collaborations

**Participation in the HP4All-3 Contributed to: **	**Yes, No. (%)**^a^
1. A clearer understanding of the urgency of a healthy and promising start	31 (83.8)
2. Intrinsic motivation to dedicate myself to enable a healthy and promising start for all children	29 (78.4)
3. Developments concerning the topic of a healthy and promising start in the municipality I am working for	29 (80.6)
4. The formulation of a clear(er) joint vision and objectives for the future	27 (75.0)
5. Easier finding/reaching professionals working in other sectors	23 (63.9)
6. New collaborations	22 (62.9)
7. Improvement of cross-sectoral collaborations	22 (61.1)

Abbreviation: HP4All-3, Healthy Pregnancy 4 All-3. ^a^ 35-37 (41%-44%) of the respondents answered the questions related to the contribution of the HP4All-3 program to the process of building cross-sectoral collaborations.

 In the free text section of the questionnaire, professionals also indicated that the HP4All-3 program provided the necessary preparatory work to accelerate the development of an action plan for cross-sectoral collaborations. Additionally, the group sessions helped to express expectations and provided an environment to connect across sectors, gain insight into the desired future, as well as to formulate concrete focus points.

## Discussion

###  Key Findings

 We identified a wide range of facilitators and barriers for building cross-sectoral collaborations. To achieve solid cross-sectoral collaborations, the following three changes are most needed in the perinatal healthcare field:

1. Structure: a solid network with a clear overview of professionals working in the different sectors needs to be built. This will help to overcome fragmentation and facilitates finding partners;
2. Culture: a joint vision/goal must be drawn up within the defined network. This will create a starting point for formulating joint actions;
3. Practice: preconditions for successful cross-sectoral collaborations are short lines of communication together with timely sharing of information.

Our research showed that there are numerous barriers for building cross-sectoral collaborations. Participation in action research offers municipalities a temporary structure for building a network. Our team helped to connect and motivate professionals working in different sectors. Additionally, action research can be helpful in creating a sense of urgency and formulating a joint vision and goal.

###  Strengths and Limitations 

 The mixed method approach of this study resulted in rich data on cross-sectoral collaboration among a wide range of professionals. Through interviews, we generated new insights on cross-sectoral collaboration and fueled the urgency for building cross-sectoral collaborations to stimulate a change process at the local level. When interpreting our results, several limitations should be taken into account. We aimed to include representatives from the local government, medical, social, and public health sector in our study. Although we approached professionals working in all these sectors in each municipality, we were unable to interview professionals from the social sector in three municipalities. Participating professionals working in the social sector often indicated that perinatal health did not concern their field of work or explained that they were not interested in an interview. We consider this an important finding because it shows that the social sector is insufficiently involved in the approach of tackling perinatal health inequities. Additionally, selection bias may have occurred as professionals with great affinity for perinatal health inequities are probably more likely to have participated in our research. However, we believe that these are exactly the professionals who are needed to initiate and build cross-sectoral collaborations. Through participation in our research they may have obtained additional tools to enthuse and motivate others to collaborate.

###  Interpretation and Practical Implications

 Our findings regarding facilitators and/or barriers of cross-sectoral collaboration to address perinatal health inequities are consistent with studies conducted in other sectors and/or countries.^42-46^ The identified barriers might originate in the large variety of backgrounds, motives, and competences of professionals working in different sectors.^23,47,48^ To overcome these barriers, change is needed in the structure, culture, and practices of the perinatal healthcare system. Our research helped to identify the necessary developments, which are outlined below.

####  Structure 

 Having a solid network is the backbone structure of well-functioning cross-sectoral collaborations. To achieve this, it is important that participating professionals are aware that there is a clear advantage to be gained by collaborating, which could not be achieved when working alone.^47^ Additionally, it is important to take psychological factors into account that can influence the formation of a strong network. During the interviews lack of trust, competitiveness, and intrinsic motivation were mentioned as factors that influence collaboration. Lack of trust can undermine collaboration: if team members doubt each other’s intentions, competence, or reliability, this can lead to the delegation of tasks and a hesitancy to share information and work together effectively. Putting one’s own ego and interests first and/or competitiveness among team members can also hinder collaboration. Although intrinsic motivation seems self-evident for successful collaboration, several factors contribute to the complexity of motivation and conviction among professionals. Professionals working in different sectors have different organizational goals, priorities, and performance metrics. Aligning these diverse objectives can be challenging, especially when there are conflicting interests. Convincing all professionals that collaboration benefits their specific goals requires careful negotiation and compromise. Additionally, collaboration requires a commitment of resources, including time, finances, and personnel. If professionals believe that the costs outweigh the benefits, it can lead to reluctance or a lack of motivation. Overcoming these impediments needs proactive efforts to foster a collaborative mindset and create a shared vision. This involves investing in relationship-building activities, setting realistic expectations, addressing concerns transparently, and actively involving all professionals in decision-making processes. Continuous evaluation and adaptation of the collaborative approach based on feedback and results are also crucial for sustaining motivation over the course of the collaboration. An external party, like the HP4All-3 research team, can be helpful to guide this process. It is important to build such a cross-sectoral network via existing networks/collaboration structures. A completely new network may lead to more fragmentation. In practice, it appears to be difficult to maintain and strengthen cross-sectoral networks without continuous support or the presence of a leading/front running professionals. To facilitate continuous support, participating municipalities of the HP4All-3 program were supported by the Dutch Centre of Expertise on Health Disparities (Pharos) after completion of the action research. This was done to ensure that the transition was further guided. Parallel to the HP4All-3 program, the national Solid Start program was launched by the Ministry of Health, Welfare, and Sport in 2018.^49^ Within Solid Start, municipalities that wish to participate receive (external) support and guidance in implementing approaches to address health inequities before, during, and after pregnancy. This can help to strengthen and expand municipal activities that were drawn up during the HP4All programs. Future research should focus on ways to perpetuate cross-sectoral collaborative initiatives.

####  Culture 

 Once the network is defined, it is important to formulate a joint vision/goal. An external party can be of great added value in this process. The HP4All-3 action research proved to be successful in creating mutual trust and expectations, inclusive participation, and a shared understanding of the problem. This resulted in collective commitment on a vision, goals, and actions. With this, the HP4All-3 program provided the right preparatory work, so that an action plan for cross-sectoral collaborations could be drawn up more quickly.

####  Practice 

 Preconditions for successful cross-sectoral collaborations are short lines of communication and timely sharing of information. To realize short lines of communication, frequent (physical) meetings are essential. By the means of a steady dialogue about communication, roles, responsibilities, and actions, professionals will gain more trust in each other and each other’s work. The past few years have been characterized by the COVID-19 pandemic that made it impossible to organize frequent physical meetings. Yet, the COVID-19 pandemic has shown that online meetings can be effective as well and make it easier for professionals to join. However, online meetings, as opposed to physical meetings, enable intensive social connection between professionals to a lesser degree. Therefore, a mix of online and physical meetings is desirable. Existing meeting structures should be used as to prevent unnecessary new meetings in already full agendas.

## Conclusion

 Our research shows that there are systemic barriers against a breakthrough in reducing perinatal health inequities. These barriers are the by-product of the current healthcare regime that is mainly focused on medical issues and organized around medical specialisms. As the determinants of perinatal health inequities are interlinked and both medical and social, new forms of collaboration across sectors are needed. This requires institutional changes to facilitate professionals to work on addressing the root causes of perinatal health inequities. Now, the incentives are rather against cross-sectoral collaboration and based on production instead of prevention. Therefore, it will require actors to come together and develop new shared visions, goals, and discourse to guide them in this transition. Applying our structure of analysis and the type of co-creative transition oriented action research can aid transformative change, as it can offer temporary structure and motivation, and help to create a sense of urgency for building cross-sectoral collaborations.

## Ethical issues

 The HP4All-3 program does not use patient data and therefore no formal ethical approval was needed for these analyses according to Dutch law.

## Competing interests

 The authors declare that they have no competing interests.

## Supplementary files


Supplementary file 1. Interview Protocol.

Supplementary file 2. Questionnaire.

Supplementary file 3. Overview of All Sub-themes Extracted Within the Facilitator “Having A Solid Network.”

Supplementary file 4. Overview of Additional Facilitators.

Supplementary file 5. Overview of Additional Barriers.
