# Supplementary material for: Building Cross-sectoral Collaborations to Address Perinatal Health Inequities: Insights From the Dutch Healthy Pregnancy 4 All-3 Program
Source: Int J Health Policy Manag. 2024 Jul 9;13:8115. doi: 10.34172/ijhpm.8115 (PMC11365078; doi:10.34172/ijhpm.8115)
Supplement: Supplementary file 1 — Interview Protocol. [file ijhpm-13-8115-s001.pdf]

Article title: Building Cross-sectoral Collaborations to Address Perinatal Health Inequities: Insights From the Dutch Healthy Pregnancy 4 All-3 Program

Journal name: International Journal of Health Policy and Management (IJHPM)

Authors' information: Leonie A. Daalderop[1,2]¶, Lisa S. Barsties[1,2]*¶, Frank van Steenbergen[2], Adja J.M. Waelput[1], Jacqueline Lagendijk[1], Jasper V. Been[1,3,4], Eric A.P. Steegers[1], Derk Loorbach[2]

[1]Department of Obstetrics and Gynaecology, Erasmus MC, University Medical Centre Rotterdam, Rotterdam, The Netherlands.

[2]Dutch Research Institute for Transitions, Erasmus University Rotterdam, Rotterdam, The Netherlands.

[3]Division of Neonatology, Department of Paediatrics, Erasmus MC – Sophia Children's Hospital, University Medical Centre Rotterdam, Rotterdam, The Netherlands.

[4]Department of Public Health, Erasmus MC, University Medical Centre Rotterdam, Rotterdam, The Netherlands.

***Correspondence to:** Lisa S. Barsties; Email: lisa.barsties@rivm.nl

Citation: Daalderop LA, Barsties LS, van Steenbergen F, et al. Building cross-sectoral collaborations to address perinatal health inequities: Insights from the Dutch Healthy Pregnancy 4 All-3 program. Int J Health Policy Manag. 2024;13:8115. doi:10.34172/ijhpm.8115

Supplementary file 1. Interview Protocol

Part 1 – 'Healthy Start' and what is being done to address health inequities in your municipality

1. What position do you hold (within your municipality)?
   a) What projects are you currently working on?
   b) What is your connection with the antenatal health care system*?
2. How would you define health inequities?
3. How would you define health inequities among pregnant women and new-borns?
4. What do you understand by the term 'Healthy Start' for children? And to what extent do you consider a 'Healthy Start' an important topic within your field of work?
   a) Has the topic 'Healthy Start', according to you, a high profile in your municipality?

5. **Civil servant/ aldermen:** To what extent are perinatal health inequities addressed in municipal policies?

   a) If health perinatal inequities are addressed in municipal policies, are such policies integrated into the general approach to health inequities? **Professional from the medical, public health, or social sector:** Are there any arrangements that pregnant women (in vulnerable situations) or women who want to conceive can make use of?

   a) To what extent are you in contact with professionals working for the municipal government concerning the care for pregnant women (in vulnerable situations) and women who want to conceive?

6. To what extent do you have insight into the scope of perinatal health inequities (e.g. perinatal mortality, preterm birth, being small for gestational age, poorer perinatal health outcomes in deprived neighbourhoods, etc.) in your municipality?

   a) To what extent do you have insight into how perinatal health inequities in your municipality relate to perinatal health inequities in surrounding cities/municipalities?

   b) To what extent are you in contact with surrounding municipalities concerning the scope of perinatal health inequities? Do you collaborate with surrounding municipalities in addressing perinatal health inequities?

Part 2 – Developments in the antenatal health care system* in relation to health inequities

7. How did the approach to perinatal health inequities develop in your municipality during the past 10 years?

   a) Has the approach to perinatal health inequities been integrated into the general approach to health inequities?

8. How did the antenatal health care system* change during the past 10 years?

9. What is necessary to decrease perinatal health inequities in your municipality in the coming years?

   a) Which developments in the antenatal health care system* are necessary to achieve this?

   b) Which elements or factors were impeding the approach to perinatal health inequities in the past?

10. Which elements or factors do you consider to be facilitators for or barriers to a future approach to perinatal health inequities?
11. What do you think can you yourself/your organisation contribute to a future approach to perinatal health inequities?
12. To address perinatal health inequities, what are the steps that need to be taken by you/your municipality in the *near future*?
13. To address perinatal health inequities, what are the steps that need to be taken by you/your municipality in the *far future*?

Part 3 – Collaborations

14. In which sector (medical, public health, social) do you consider yourself working in? How would you describe the other sectors (medical, public health, social)?
15. With whom are you collaborating (a lot) within your own sector?
    a) What does this collaboration look like and how is it going? Could you give specific examples of this collaboration?
16. **Civil servant/ aldermen:** To what extent are you collaborating with civil servants or aldermen from other fields than your own, or with other professionals from other sectors than your own (medical, public health, social)?
    a) What does this collaboration look like and how is it going? Could you give specific examples of this collaboration?

    Professional from the medical, public health, or social sector: To what extent do you collaborate with professionals from other sectors than your own (medical, public health, social)?

    b) What does this collaboration look like and how is it going? Could you give specific examples of this collaboration?
17. With whom would you like to collaborate differently/more/less? Could you give concrete examples of how you would like to change these collaborations?
18. What do you consider to be challenges for the collaboration with professionals from your own/other sectors (medical, public health, social)?
19. Which events, groups, and/or individuals do you think have a strong impact on the development of the approach to perinatal health inequities?
    a) Think of examples in your own municipality as well as in broader society.

20. What or whom do you consider an important source of inspiration for the approach to perinatal health inequities?
    a) Think of examples in your own municipality as well as in broader society.

Part 4 – Summing-up (optional)

21. Whom else within your municipality should we talk to (medical, public health, or social sector)? Who holds an interesting perspective on or can share interesting experiences within the approach to perinatal health inequities? And why?
22. Are there any documents or texts that offer an interesting perspective on the approach to perinatal health inequities within your municipality?
23. Is there anything else you would like to share with us that we have not discussed so far?
24. What would you like to get from the collaboration with us and the Healthy Pregnancy 4 All team?

*****The antenatal health care system encompasses care delivered by professionals from the medical, social, and/or public health sector for (pregnant) women and their (unborn) child(ren) before, during, and after pregnancy.
