# Supplementary material for: Building Cross-sectoral Collaborations to Address Perinatal Health Inequities: Insights From the Dutch Healthy Pregnancy 4 All-3 Program
Source: Int J Health Policy Manag. 2024 Jul 9;13:8115. doi: 10.34172/ijhpm.8115 (PMC11365078; doi:10.34172/ijhpm.8115)
Supplement: Supplementary file 2 — Questionnaire. [file ijhpm-13-8115-s002.pdf]

Article title: Building Cross-sectoral Collaborations to Address Perinatal Health Inequities: Insights From the Dutch Healthy Pregnancy 4 All-3 Program

Journal name: International Journal of Health Policy and Management (IJHPM)

Authors' information: Leonie A. Daalderop[1,2]¶, Lisa S. Barsties[1,2]*¶, Frank van Steenbergen[2], Adja J.M. Waelput[1], Jacqueline Lagendijk[1], Jasper V. Been[1,3,4], Eric A.P. Steegers[1], Derk Loorbach[2]

[1]Department of Obstetrics and Gynaecology, Erasmus MC, University Medical Centre Rotterdam, Rotterdam, The Netherlands.

[2]Dutch Research Institute for Transitions, Erasmus University Rotterdam, Rotterdam, The Netherlands.

[3]Division of Neonatology, Department of Paediatrics, Erasmus MC – Sophia Children's Hospital, University Medical Centre Rotterdam, Rotterdam, The Netherlands.

[4]Department of Public Health, Erasmus MC, University Medical Centre Rotterdam, Rotterdam, The Netherlands.

***Correspondence to:** Lisa S. Barsties; Email: lisa.barsties@rivm.nl

Citation: Daalderop LA, Barsties LS, van Steenbergen F, et al. Building cross-sectoral collaborations to address perinatal health inequities: Insights from the Dutch Healthy Pregnancy 4 All-3 program. Int J Health Policy Manag. 2024;13:8115. doi:10.34172/ijhpm.8115

Supplementary file 2. Questionnaire

Part 1 – Introduction

1. Which municipality are you working in? In case you are working in the municipality of Heerlen *and* the municipality of Landgraaf, please select only one of them.
   a) Den Haag
   b) Eemsdelta
   c) Heerlen
   d) Landgraaf
   e) Vlissingen
   f) Enschede
2. What activities of the HP4All-3 program have you participated in? You can select several answers.

a) Interview

b) Group session 1 (future systemic shifts, ‘from-to’ exercise)

c) Group session 2 (formulating actions, X-curve exercise, prioritizing actions) d) I don't know

3. Which occupational group do you belong to?

a) Midwife (1st tier)

b) Midwife/gynaecologist (2nd tier)

c) Maternity care

d) General practitioner

e) Preventive Child Health Care

f) Youth care

g) Safe Home

h) Paediatrics

i) Municipality: Civil servant

j) Municipality: Alderman

k) Municipality: Neighbourhood team

l) Welfare

m) Education

n) Municipal Health Services

o) Other:

4. What is your profession?

----

5. How often do you professionally engage in the topic healthy and promising start? a) Daily

b) Weekly

c) Monthly

d) Rarely to never

If answers a, b, or c were selected, please go to item 6; if answer d was selected, please go to item 7.

6. Please describe the activities with regards to the topic healthy and promising start you engage in. ---

7. How often are you in contact with couples who wish to become parents, pregnant women, and/or young families?

a) Daily
b) Weekly
c) Monthly
d) Rarely to never

Part 2 – Collaborations

8. Currently, I am working with the following occupational groups; see occupational groups item 3, answers a-m; for each occupational group the following answers are possible:

a) Yes, and the collaboration is going well
b) Yes, but I would like to collaborate more
c) No, but I would like to collaborate
d) No, but collaboration is not necessary

If answer b was selected, please go to item 9; if answer c was selected, please go to item 10; if answers a or d were selected, please go to item 11.

9. You indicated that you already collaborate with professionals from the occupational group X, but that you would like to collaborate more. What is hindering more collaboration? You can select several answers.

a) Not enough time
b) Not enough trust in each other
c) I do not know whom to work with from the occupational group X
d) It is not clear what the occupational group X can do for me
e) Other:

10. You indicated that you would like to collaborate with professionals from the occupational category X. Why do you think that you are not yet collaborating? You can select several answers. a) Not enough time

b) Not enough trust in each other

c) I do not know whom to work with from the occupational group X

d) It is not clear what the occupational group X can do for me e) Other:

11. I can find/reach professionals/organizations from the following occupational groups; see occupational groups item 3, answers a-m; for each occupational group the following answers are possible:

a) Strongly disagree
b) Disagree
c) Agree
d) Strongly agree
e) I don't know
f) Not necessary/relevant

If answers a or b were selected, please go to item 12; if answers c, d, or e were selected, please go to item 13.

12. You indicated that you cannot find/reach professionals/organizations from the occupational category X. Why? You can select several answer categories.

a) There is no overview of involved professionals/organizations from the occupational group X
b) I don't have enough time to find out more about involved professionals/organizations from the occupational group X
c) There is no clear point of contact for the occupational group X
d) The procedure of meetings is unclear
e) Professionals/organizations from the occupational group X are hard to reach
f) I don't understand the structure and practices of the occupational group X g) Other:

Part 3 – Policy

13. Municipal policies for a healthy and promising start focus on (becoming) families in the following periods. You can select several answers.

a) The period before pregnancy (preconceptional)
b) Pregnancy
c) Maternity period
d) The first year of life of the child(ren)
e) The second year of life of the child(ren)

f) The third year of life of the child(ren
g) The fourth year of life of the child(ren)
h) There is no policy focusing on any of these periods
i) I don’t know

14. A healthy and promising start is on the municipal agenda.
 a) Strongly disagree
 b) Disagree
 c) Neither agree nor disagree
 d) Agree
 e) Strongly agree
 f) I don’t know

15. Which municipal policy domains engage in the topic healthy and promising start? You can select several answers.
 a) Youth
 b) Public health
 c) Welfare
 d) Work and income
 e) Health
 f) I don’t know
 g) Other

16. Do you or your colleagues get involved in the drawing up of municipal policy plans with regard to a healthy and promising start?
 a) Yes
 b) Yes, but we would like to get involved more
 c) No, but we would like to get involved
 d) No, but that is not necessary
 e) I don’t know

 If answers b or c were selected, please go to item 17; if answers a, d, or e were selected, please go to item 18.

17. You indicated that you and/or your colleagues don’t get involved (enough) in the drawing up of municipal policy plans with regard to a healthy and promising start. Why do you think this is? You can select several answers.

a) There is not enough communication between the municipality and my occupational group
b) The municipality does not know enough about the topic healthy and promising start and therefore does not know whom to involve
c) Civil servants do not have enough time to meet professionals from my occupational group
d) My occupational group is not known well enough by the municipality
e) My occupational group does not have a clear point of contact at the municipality
f) Other:

Part 4 – Impact of HP4All-3

18. Participating in HP4All-3 contributed to a clearer understanding of the urgency of a healthy and promising start.
    a) Strongly disagree
    b) Disagree
    c) Neither agree nor disagree
    d) Agree
    e) Strongly agree
    f) I don’t know
19. Participating in HP4All-3 increased my intrinsic motivation to dedicate myself to enable a healthy and promising start for all children.
    a) Strongly disagree
    b) Disagree
    c) Neither agree nor disagree
    d) Agree
    e) Strongly agree
    f) I don’t know
20. Participating in HP4All-3 contributed to developments concerning the topic of a healthy and promising start in the municipality I am working in.
    a) Strongly disagree
    b) Disagree
    c) Neither agree nor disagree

d) Agree
e) Strongly agree
f) I don’t know

If answers a or b were selected, please go to item 21, then 23; if answers d or e were selected, please go to item 22; if answers c or f were selected, please go to item 23.

21. Please explain why HP44All-3 did not contribute to developments concerning the topic of a healthy and promising start in the municipality you are working in?

----

22. Please explain why HP4All-3 contributed to developments concerning the topic of a healthy and promising start in the municipality you are working in?

----

23. Participating in HP4All-3 contributed to the formulation of a clear(er) joint vision and objectives for the future.

a) Strongly disagree
b) Disagree
c) Neither agree nor disagree
d) Agree
e) Strongly agree
f) I don’t know

If answers a or b were selected, please go to item 24, then 26; if answers d or e were selected, please go to item 25; if answers c or f were selected, please go to item 26.

24. How could HP4All-3 have contributed to the formulation of a clear(er) joint vision and objectives for the future? You can select several answers.

a) A more diverse group of participants at the group sessions
b) More guidance by the researchers during the group sessions
c) More concrete advice by the researchers
d) More group sessions
e) I don’t know
f) Other:

25. Which elements of HP4All-3 contributed to formulation of a clear(er) joint vision and objectives for the future? You can select several answers.

a) Getting together with other professionals during the group sessions
b) The presence of an independent party during the group sessions
c) Brainstorming about the problem (group session 1)
d) Getting a clear overview of the existing network (group session 1)
e) Thinking about future systemic shifts (group session 1)
f) Formulating actions/X-curve exercise (group session 2)
g) Prioritizing actions (group session 2)
h) Thinking about roles and one's personal responsibility (group session 2)
i) The action agenda
j) I don't know
k) Other:

26. Participating in HP4All-3 helped me to get an (even) better overview of the professionals/organizations that are involved in the topic healthy and promising start in my municipality.
a) Yes
b) No, I already a had a good overview
c) No, HP4All-3 did not offer any new insights
d) I don't know

27. Participating in HP4All-3 helped me to find/reach professionals from other occupational groups more easily.
a) Strongly disagree
b) Disagree
c) Neither agree nor disagree
d) Agree
e) Strongly agree

28. Participating in HP4All-3 contributed to new collaborations.
a) Strongly disagree
b) Disagree
c) Neither agree nor disagree
d) Agree
e) Strongly agree
f) I don't know

29. Participating in HP4All-3 improved existing collaborations with professionals from other occupational groups.

a) Strongly disagree
b) Disagree
c) Neither agree nor disagree
d) Agree
e) Strongly agree
f) I don’t know

30. Did you miss professionals/organizations at the group sessions?

a) No
b) Yes
c) I don’t know
d) I did not participate in the group sessions

If answer b was selected, please go to item 31, then 31; if answers a, c, or d were selected, please go to item 32.

31. Name the professionals/organizations that you missed at the group sessions.

----

32. Participating in HP4All-3 led to …. You can select several answers.

a) Converting existing activities
b) Building up new activities
c) Phasing out existing activities
d) Building on existing activities
e) None of these
f) I don’t know

If you selected answer a, please go to item 33; if you selected answer b, please go to item 34; if you selected answer c, please go to item 35; if you selected answer d, please go to item 36; if you selected answer e or f, please go to item 37.

33. Shortly describe the converted activities.

----

34. Shortly describe the built up activities.

----

35. Shortly describe the phased out activities. ---
----

36. Shortly describe activities you built on.

----

37. Which occupational group is the initiator for the converted, built up, phased out, or built on activities? You can select several answers.

a) Midwife (1st tier)
b) Midwife/gynaecologist (2nd tier)
c) Maternity care
d) General practitioner
e) Preventive Child Health Care/Municipal Health Services
f) Youth care
g) Safe Home
h) Paediatrics
i) Municipality: Civil servant
j) Municipality: Alderman
k) Municipality: Neighbourhood team
l) Welfare
m) Education
n) Other:

38. Following the group sessions, you received an action-agenda, including the results of our research. The information given in the action-agenda was/is being used for the local healthy and promising start approach.

a) Strongly disagree
b) Disagree
c) Neither agree nor disagree
d) Agree
e) Strongly agree
f) I don’t know

Part 5 – Summing up

39. Would you like to share anything else with regards to a healthy and promising start or your participating in HP4All-3?
