# Supplementary material for: Building Cross-sectoral Collaborations to Address Perinatal Health Inequities: Insights From the Dutch Healthy Pregnancy 4 All-3 Program
Source: Int J Health Policy Manag. 2024 Jul 9;13:8115. doi: 10.34172/ijhpm.8115 (PMC11365078; doi:10.34172/ijhpm.8115)
Supplement: Supplementary file 3 — Overview of All Sub-themes Extracted Within the Facilitator “Having A Solid Network.” [file ijhpm-13-8115-s003.pdf]

Article title: Building Cross-sectoral Collaborations to Address Perinatal Health Inequities: Insights From the Dutch Healthy Pregnancy 4 All-3 Program

Journal name: International Journal of Health Policy and Management (IJHPM)

Authors' information: Leonie A. Daalderop[1,2]¶, Lisa S. Barsties[1,2]*¶, Frank van Steenbergen[2], Adja J.M. Waelput[1], Jacqueline Lagendijk[1], Jasper V. Been[1,3,4], Eric A.P. Steegers[1], Derk Loorbach[2]

[1]Department of Obstetrics and Gynaecology, Erasmus MC, University Medical Centre Rotterdam, Rotterdam, The Netherlands.

[2]Dutch Research Institute for Transitions, Erasmus University Rotterdam, Rotterdam, The Netherlands.

[3]Division of Neonatology, Department of Paediatrics, Erasmus MC – Sophia Children's Hospital, University Medical Centre Rotterdam, Rotterdam, The Netherlands.

[4]Department of Public Health, Erasmus MC, University Medical Centre Rotterdam, Rotterdam, The Netherlands.

***Correspondence to:** Lisa S. Barsties; Email: lisa.barsties@rivm.nl

Citation: Daalderop LA, Barsties LS, van Steenbergen F, et al. Building cross-sectoral collaborations to address perinatal health inequities: Insights from the Dutch Healthy Pregnancy 4 All-3 program. Int J Health Policy Manag. 2024;13:8115. doi:10.34172/ijhpm.8115

Supplementary file 3. Overview of All Sub-themes Extracted Within the Facilitator "Having A Solid Network."

Facilitator	***Category***	***Description***
Knowing and/or finding each other	Practical	Knowing potential collaboration partners and being able to find them enables cross-sectoral collaboration. 'Knowing' many professionals means literally having seen (the face of) other professionals. An overview of professionals working in different sectors, including their contact details, is a good starting point. Respondents also indicated that trusting professionals from other sectors is easier when

		they know them. Finally, only if you know professionals from other sectors, it is possible to “use” their skills and expertise.
Meeting other professionals	Practical	(Physically) getting together helps to create new ideas, knowing each other’s skills and expertise, and identifying common goals.
Being familiar with each other’s work	Practical	Knowing what and how other professionals are working (on) helps to make agreements across sectors. Only if you are familiar with each other’s work, you can approach the right professional when in need of help.
Joining forces	Practical	To collaborate across sectors, professionals need to be motivated to join forces. Also, all professionals need to feel responsible for the problem at stake.
Involving each other	Practical	Informing colleagues from other sectors about what you are working on, as well as involving them in your activities is important for building cross-sectoral collaborations. When working together on a project, it can be helpful to do so from the beginning onwards.
Connector	Structural	A professional/organization that knows professionals/organizations from across different sectors, as well as their skills, expertise, needs, and structures is able to connect sectors. A connector can also motivate others to collaborate. This can be any professional.
Division of tasks	Structural	Knowing one’s tasks and responsibilities enables smooth collaboration across sectors.

Meeting structure	Structural	Meeting professionals from different sectors on a regular basis helps to create and keep an overview of activities and changes across sectors.
Reinforcing each other	Cultural	Knowing one's and each other's abilities and responsibilities as well as not wanting to compete is crucial in working across sectors.
Joint dossier	Structural	It is helpful to work with a joint dossier in order to know which services other professionals have offered a client already. This can prevent repeated actions and thereby save time.
Infrastructure	Structural	An infrastructure in which collaboration across sectors is an integral, pre-defined, and outlined part helps to find and work with professionals from other sectors.
