# Supplementary material for: Building Cross-sectoral Collaborations to Address Perinatal Health Inequities: Insights From the Dutch Healthy Pregnancy 4 All-3 Program
Source: Int J Health Policy Manag. 2024 Jul 9;13:8115. doi: 10.34172/ijhpm.8115 (PMC11365078; doi:10.34172/ijhpm.8115)
Supplement: Supplementary file 4 — Overview of Additional Facilitators. [file ijhpm-13-8115-s004.pdf]

Article title: Building Cross-sectoral Collaborations to Address Perinatal Health Inequities: Insights From the Dutch Healthy Pregnancy 4 All-3 Program

Journal name: International Journal of Health Policy and Management (IJHPM)

Authors' information: Leonie A. Daalderop[1,2]¶, Lisa S. Barsties[1,2]*¶, Frank van Steenbergen[2], Adja J.M. Waelput[1], Jacqueline Lagendijk[1], Jasper V. Been[1,3,4], Eric A.P. Steegers[1], Derk Loorbach[2]

[1]Department of Obstetrics and Gynaecology, Erasmus MC, University Medical Centre Rotterdam, Rotterdam, The Netherlands.

[2]Dutch Research Institute for Transitions, Erasmus University Rotterdam, Rotterdam, The Netherlands.

[3]Division of Neonatology, Department of Paediatrics, Erasmus MC – Sophia Children's Hospital, University Medical Centre Rotterdam, Rotterdam, The Netherlands.

[4]Department of Public Health, Erasmus MC, University Medical Centre Rotterdam, Rotterdam, The Netherlands.

***Correspondence to:** Lisa S. Barsties; Email: lisa.barsties@rivm.nl

Citation: Daalderop LA, Barsties LS, van Steenbergen F, et al. Building cross-sectoral collaborations to address perinatal health inequities: Insights from the Dutch Healthy Pregnancy 4 All-3 program. Int J Health Policy Manag. 2024;13:8115. doi:10.34172/ijhpm.8115

Supplementary file 4. Overview of Additional Facilitators

Facilitator	***Category***	***Description***
Trust	Cultural	Many professionals described that trust in each other's working methods, having an open attitude towards each other, and being able to talk openly about all kinds of subjects, positively affect cross-sectoral collaborations.
Common language	Cultural	Understanding what professionals from other sectors are talking about can be challenging. Creating a common language or learning how to understand each other's language is an

		important prerequisite for cross-sectoral collaborations.
Support base	Structural	When collaborating across sectors, it is important that there is a strong support base in every sector, preferably at every organizational level (politics, management, and execution). To create such a support base, it is essential that all involved professionals have the same sense of urgency.
Money	Structural	Shared finances can help to organize care more efficiently. Additionally, financial support can enable the funding of necessary collaboration structures, platforms, and communication.
