# Supplementary material for: Building Cross-sectoral Collaborations to Address Perinatal Health Inequities: Insights From the Dutch Healthy Pregnancy 4 All-3 Program
Source: Int J Health Policy Manag. 2024 Jul 9;13:8115. doi: 10.34172/ijhpm.8115 (PMC11365078; doi:10.34172/ijhpm.8115)
Supplement: Supplementary file 5 — Overview of Additional Barriers. [file ijhpm-13-8115-s005.pdf]

Article title: Building Cross-sectoral Collaborations to Address Perinatal Health Inequities: Insights From the Dutch Healthy Pregnancy 4 All-3 Program

Journal name: International Journal of Health Policy and Management (IJHPM)

Authors' information: Leonie A. Daalderop[1,2]¶, Lisa S. Barsties[1,2]*¶, Frank van Steenbergen[2], Adja J.M. Waelput[1], Jacqueline Lagendijk[1], Jasper V. Been[1,3,4], Eric A.P. Steegers[1], Derk Loorbach[2]

[1]Department of Obstetrics and Gynaecology, Erasmus MC, University Medical Centre Rotterdam, Rotterdam, The Netherlands.

[2]Dutch Research Institute for Transitions, Erasmus University Rotterdam, Rotterdam, The Netherlands.

[3]Division of Neonatology, Department of Paediatrics, Erasmus MC – Sophia Children's Hospital, University Medical Centre Rotterdam, Rotterdam, The Netherlands.

[4]Department of Public Health, Erasmus MC, University Medical Centre Rotterdam, Rotterdam, The Netherlands.

***Correspondence to:** Lisa S. Barsties; Email: lisa.barsties@rivm.nl

Citation: Daalderop LA, Barsties LS, van Steenbergen F, et al. Building cross-sectoral collaborations to address perinatal health inequities: Insights from the Dutch Healthy Pregnancy 4 All-3 program. Int J Health Policy Manag. 2024;13:8115. doi:10.34172/ijhpm.8115

Supplementary file 5. Overview of Additional Barriers

Barrier	***Category***	***Description***
Unclear responsibilities	Practical	Many professionals described unclear responsibilities as well as the lack of shared responsibilities to address perinatal health inequities as major barriers for cross-sectoral collaborations.
Privacy regulations	Structural	Professionals experienced the General data Protection Regulation (GDFR) as a barrier for cross-sectoral collaborations as they do not know which information can be shared. This

		gives them a feeling of insecurity and fear when they would like to share information.
Lack of a common goal/plan	Structural	Many professionals described that the lack of a common goal/plan between sectors is a barrier for efficient and effective cross-sectoral collaborations.
Too many involved professional/organizations	Structural	Professionals mentioned that the magnitude of organizations involved in the topic of perinatal health (e.g., hospitals, welfare organizations, maternity care organizations, and neighbourhood teams) hinders efficient and effective crosssectoral collaborations.
Insufficient and discontinuous capacity	Practical	Respondents complained about frequent personnel changes. These are at the expense of continuity and hinder collaboration. Another problem is that teams often are composed of only a few professionals. If someone leaves the team, there is an immediate understaffing, which has a direct impact on collaboration structures.
